# Comparative Efficacy of MultiModal AI Methods in Screening for Major Depressive Disorder: Machine Learning Model Development Predictive Pilot Study

**DOI:** 10.2196/56057

**Published:** 2025-05-30

**Authors:** Donghao Chen, Pengfei Wang, Xiaolong Zhang, Runqi Qiao, Nanxi Li, Xiaodong Zhang, Honggang Zhang, Gang Wang

**Affiliations:** 1 School of Artificial Intelligence, Beijing University of Posts and Telecommunications, Beijing, China; 2 Beijing Key Laboratory of Mental Disorders, National Clinical Research Center for Mental Disorders & National Center for Mental Disorders, Beijing Anding Hospital, Capital Medical University, Beijing, China; 3 Advanced Innovation Center for Human Brain Protection, Capital Medical University, Beijing, China

**Keywords:** major depressive disorder, artificial intelligence, computational psychiatry, facial action unit, multimodal analysis, multiparadigm analysis, MDD

## Abstract

**Background:**

Conventional approaches for major depressive disorder (MDD) screening rely on two effective but subjective paradigms: self-rated scales and clinical interviews. Artificial intelligence (AI) can potentially contribute to psychiatry, especially through the use of objective data such as objective audiovisual signals.

**Objective:**

This study aimed to evaluate the efficacy of different paradigms using AI analysis on audiovisual signals.

**Methods:**

We recruited 89 participants (mean age, 37.1 years; male: 30/89, 33.7%; female: 59/89, 66.3%), including 41 patients with MDD and 48 asymptomatic participants. We developed AI models using facial movement, acoustic, and text features extracted from videos obtained via a tool, incorporating four paradigms: conventional scale (CS), question and answering (Q&A), mental imagery description (MID), and video watching (VW). Ablation experiments and 5-fold cross-validation were performed using two AI methods to ascertain the efficacy of paradigm combinations. Attention scores from the deep learning model were calculated and compared with correlation results to assess comprehensibility.

**Results:**

In video clip-based analyses, Q&A outperformed MID with a mean binary sensitivity of 79.06% (95%CI 77.06%‐83.35%; *P*=.03) and an effect size of 1.0. Among individuals, the combination of Q&A and MID outperformed MID alone with a mean extent accuracy of 80.00% (95%CI 65.88%‐88.24%; *P*= .01), with an effect size 0.61. The mean binary accuracy exceeded 76.25% for video clip predictions and 74.12% for individual-level predictions across the two AI methods, with top individual binary accuracy of 94.12%. The features exhibiting high attention scores demonstrated a significant overlap with those that were statistically correlated, including 18 features (all *Ps*<.05), while also aligning with established nonverbal markers.

**Conclusions:**

The Q&A paradigm demonstrated higher efficacy than MID, both individually and in combination. Using AI to analyze audiovisual signals across multiple paradigms has the potential to be an effective tool for MDD screening.

## Introduction

Depressive disorder is a common mental disorder affecting approximately 322 million patients in the world, with major depressive disorder (MDD) as one of its two main subcategories, which can significantly affect all aspects of life, including performance at school, productivity at work, and relationships with family and friends [1]. The primary methods to assess depression encompass mental status examinations and assessment scales. However, mental status examinations such as the Hamilton Rating Scale for Depression (HAMD) necessitate direct, in-person interviews conducted by clinicians, which can result in processes that are both time-consuming and labor-intensive [2]. Self-Report Symptom Inventories (SRSI) such as the Beck Depression Inventory [3] and the Patient Health Questionnaire-9 (PHQ-9) [4] are time-efficient but can be influenced by subjective biases, which allows for individual variability [5]. Therefore, the outcomes are susceptible to both intentional and unintentional subjective influences [6] and more approaches are needed to improve efficiency and accuracy.

In recent years, artificial intelligence (AI) has garnered attention for its application in signal analysis across various modalities. For instance, support vector machines have been used to analyze functional magnetic resonance imaging (fMRI) data [7] and a convolutional neural network (CNN) has been applied to an electroencephalogram (EEG) [8] to detect depression. While physiological signals such as fMRI and EEG are unaffected by subjective factors and directly reflect the participants’ physical states, they involve complex procedures and high costs. In contrast, noncontact signals, including text, audio, visual content, and scale information are more accessible for analysis.

In the text modality, hidden Markov models and random forest models were developed to predict depression and posttraumatic stress disorder based on frequency of Twitter usage and content [9]. By aggregating weighted words using lexicons, the sentiment score derived from text messages demonstrated a positive association with the severity of depression as measured by the self-rated Patient Health Questionnaire-8 (PHQ-8) [10-12]. Among the audio modalities, speech patterns such as a narrowed pitch range and reduced phonemes within the vowel space have emerged as important objective indicators for assessing depressive states [13,14]. Along with prosodic features, mel frequency cepstral coefficients (MFCC) [15], detailed spectral features [16], and deep-learned acoustic characteristics [17] have also been used to identify the presence of depressive symptoms, achieving binary accuracy of up to 79% or *F*_1_-score of 0.890.

For the visual or multimodal domain, several open datasets are available. One notable example is the Audio/Visual Emotion Challenge and Workshop [18], which focuses on the detection of depression and uses an audio-visual dataset that includes image features extracted from original images and audio recordings and transcribed text from Google Cloud, paired with the PHQ-8 scores. Facial action units (AU), as outlined in the Facial Action Coding System [19], serve as the foundation for facial expressions and constitute essential image features in the Audio/Visual Emotion Challenge and Workshop. Commonly observed AUs correspond to a range of expressions such as smiling and frowning (see Table S1 in Multimedia Appendix 1). A higher overall frowning (Action Unit 4, ie AU4) and head-down posture were identified in a study by Fiquer et al [20], while a lower overall AU12 and a markedly higher overall AU14 were identified in a study by Girard et al [21]. This indicated that the distribution of AUs differs significantly between depressed and nondepressed persons. Facial Action Coding System has also been employed in the analysis of stress [22], anxiety [23], and Parkinson’s disease [24].

There are several other existing datasets, including Mundt-35 [25], BlackDog [26], and MODMA [27]. Most of these datasets contain a single paradigm, primarily relying on interviews such as HAMD, or targeting the scores of SRSIs such as the PHQ-8. Additionally, current multimodal AI methods mainly extract local features from utterances or sentences for video clip predictions [28,29]. At the same time, we believe the screening and diagnosis of MDD should include the entire process, similarly to the process of clinical practice.

For other paradigm options, mental imagery description (MID) [30] can manifest across different sensory modalities, encompassing visual [31,32], auditory [33], and textual information, and tends to evoke stronger emotional responses than verbal processing [30].

Thus, aiming to evaluate the efficacy of different paradigms, we aggregated them in a tool, namely the Electronic Tool for Depression (ETD), and used a state-of-the-art (SOTA) method using audiovisual signals to validate their efficacies. We propose the ETD to be a nonsubjective and easy-use MDD screening tool. The SOTA method generates predictions on video clips, and two of the four paradigms contain only visual signals; therefore, we implemented a voting mechanism for individual predictions and proposed a global feature method for the remaining vision-only paradigms. This pilot study underscores our primary contributions, which can be summarized as follows: (1) to validate the efficacy of the paradigms via AI on audiovisual signals and aggregate them within a tool for MDD screening and (2) to propose a global feature method and explore its efficacy and interpretability.

## Methods

### Design of the Task and Building the Tool

**Figure 1.**
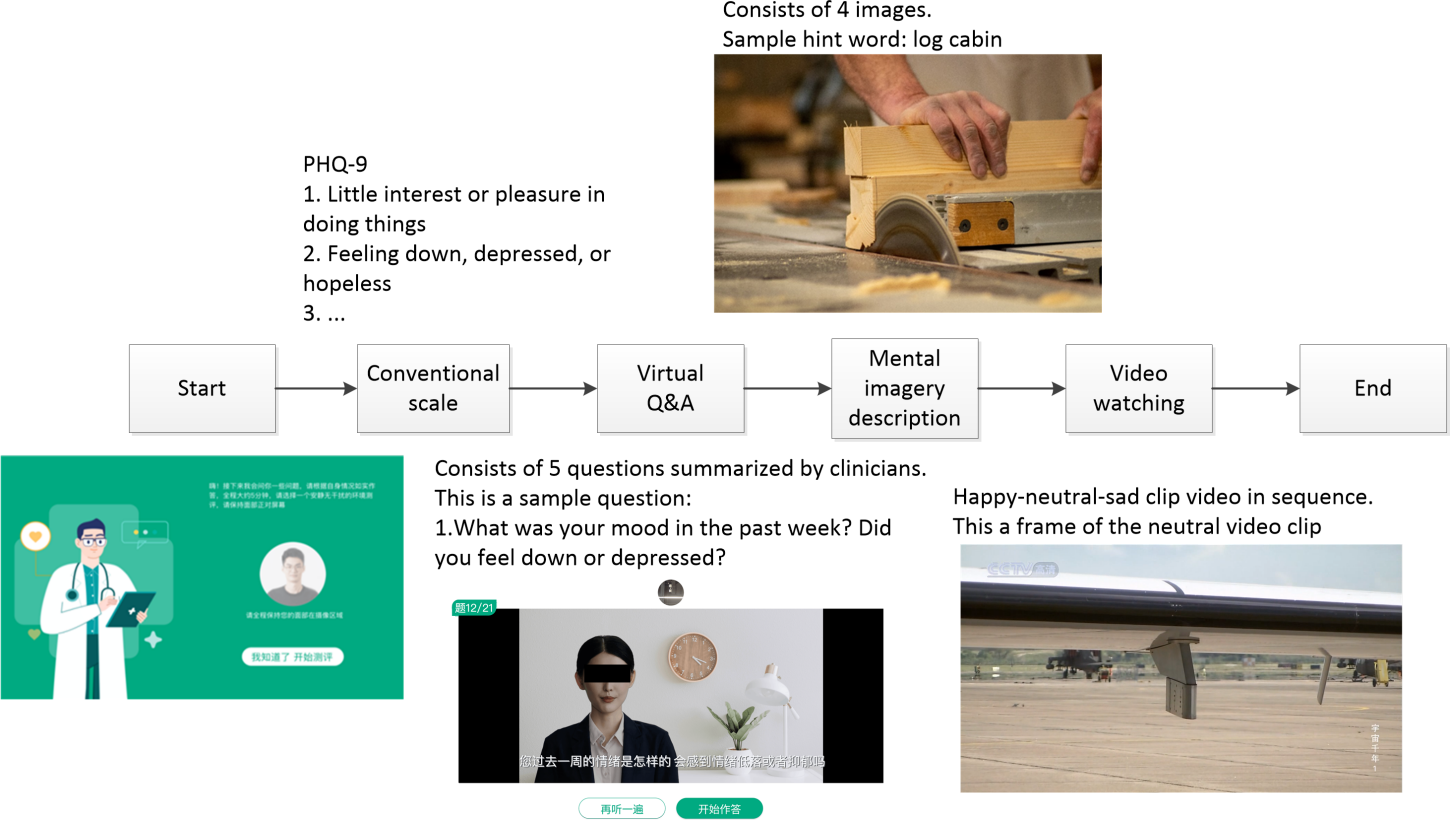
Components of the Electronic Tool for Depression (ETD). PHQ-9: Patient Health Questionnaire-9; Q&A: question-and-answer.

The ETD consists of four paradigms, aggregated into an application designed for an 11.5-inch tablet featuring an 8-MP front-facing camera and a 44.8 kHz sample rate microphone. Before using the ETD, clinicians adjusted the tablet to ensure that the participant’s head is aligned with the device at an appropriate distance (approximately 50 centimeters) for effective face capture. The ETD structure and app design are depicted in Figure 1. Paradigm 1 uses a conventional self-rated scale, specifically the PHQ-9. Paradigm 2 encompasses a question-and-answering (Q&A) paradigm simulating psychiatric examinations. Paradigm 3 requires participants to describe images with the hint words [30-33]. Paradigm 4 presents three video clips of varying emotional sentiment scores [34,35] in a positive, neutral, and negative sequence (41‐73 seconds, average 60.33 seconds). Participants sequentially respond to these components, with recordings capturing their reactions during both the viewing and responding phases, including the PHQ-9 selections; the entire process takes approximately 5 minutes. It is essential to clarify that the scale was only used to elicit reactions, and its scores did not contribute to the predictions.

### Recruitment

In this study, 89 participants were recruited from April 2022 to December 2022 in Beijing. Among these, 51 were recruited from the Beijing Anding Hospital Inpatient Department and were all diagnosed with MDD by experienced psychiatrists according to the International Code of Diseases, tenth revision (*ICD-10*) [36]. All participants met the inclusion criteria, which were as follows: (1) age 18‐65 years, (2) proficiency in standard Chinese, (3) educational level of primary school or above, and (4) ability to understand and cooperate with the research protocol.

Exclusion criteria included (1) diagnosis of schizophrenia, schizoaffective disorder, or other mental disorders and (2) history of organic brain disease. The remaining 38 participants were recruited openly from the general population (employees and college students) who were not experiencing depression-related symptoms.

Participants from the hospital completed two steps: the first involved using the ETD app, and the second included assessment using the HAMD-17 scale by clinicians. Community participants only completed the ETD test, and all were confirmed to have no depressive symptoms based on the PHQ-9 assessment. Finally, the asymptomatic and the healthy control groups formed the nonMDD group (48 participants), while the mild group and the moderate or severe group were collectively referred to as the MDD group (41 participants). For ease of explanation, the mild group was designated as MDD-sub1, and the moderate or severe group was designated as MDD-sub2. Sex was compared using the *Χ*^*2*^ test; age and HAMD scores were compared using the Mann-Whitney *U* test. There were no significant differences in sex ratio or age between the groups, while the MDD group had significantly higher PHQ-9 scores than the nonMDD group.

### Ethical Considerations

This study was approved by the Ethics Committee of Beijing Anding Hospital Capital Medical University. No compensation fee was paid to participants, with written informed consent obtained for the data usage of research analysis. Data were deidentified and all analyses followed data privacy guidelines.

### Model Training

**Figure 2.**
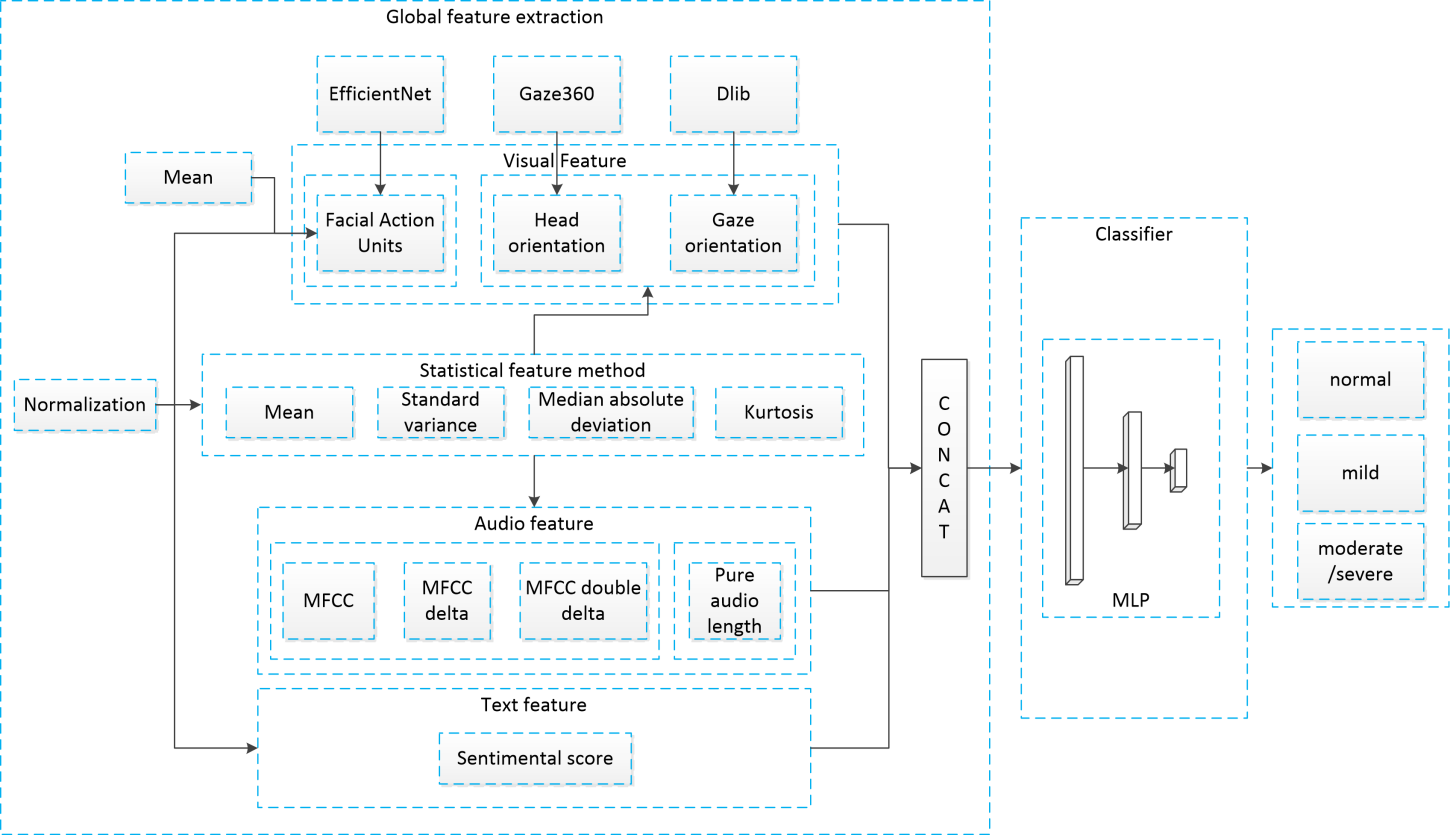
The global feature extraction method architecture. MFCC: mel frequency cepstral coefficients; MLP: multilayer perceptron.

All recorded videos underwent a manual verification process to ensure that the image ratio of a complete head, face, and eyes exceeded the empirical 95% threshold. We adopted the MFCC-based recurrent neural network (RNN) [29] as the validation model, which used a multimodal method that integrated MFCC and AU features and achieved a SOTA accuracy of 95.6% in binary classification of depression on the DAIC-WOZ (Distress Analysis Interview Corpus) dataset [18]. We pretrained the RNN on RAVDESS (Ryerson Audio-Visual Database of Emotional Speech and Song dataset) [37], aggregated the AU features, and fine-tuned the model on our dataset. We developed a CNN model for AU detection using EfficientNet [38] on BP4D [39], achieving a mean *F*_1_-score of 0.76 on selected AUs. The sample size of video clips for the RNN was 11,075, comprising 6826 normal, 2933 mild, and 1316 moderate or severe instances. We established a clip-voting ratio to represent the individual results. While the RNN simultaneously processed local audio and visual data, it did not incorporate conventional scale and video watching. To address this limitation, we proposed a global feature extraction method (depicted in Figure 2) to derive global features and build AI models. For the vision modality, we used Gaze360 [40] and Dlib [41] along with AU features to estimate gaze and head orientation. For the audio modality, we extracted MFCC-based features and the pure audio duration of the human voice. For the text modality, we calculated sentiment scores using the *pyltp* package [42]. The features were concatenated in the order of visual, audio, and text features. Additionally, we incorporated statistical characteristics such as mean and variance to enhance their global representation. Normalization and bias adjustments were applied to ensure that all the features were positive for later attention computation (complete feature list is provided in Table S2 in Multimedia Appendix 1).

We adopted a multilayer perceptron (MLP) as our classifier for global predictions, which is identical to the RNN. The MLP comprises layers with 512, 1024, 128, and 3 neurons, incorporating batch normalization and a 0.2 dropout rate to mitigate overfitting; a softmax layer was added as the prediction. The Adam optimizer was used with a base learning rate of 1e-3, β_1_ of 0.9, β_2_ of 0.999, and ε of 1e-8. Given that deep learning methods are often considered “dark magic,” we sought to enhance comprehensibility by employing Grad-Cam [43] to visualize the attention scores of the MLP’s best-performing model across each feature. These results were then compared with Spearman and Kendall correlation coefficients computed using scikit-learn [44].

### Statistical Analysis

Ablation experiments were conducted on various paradigm combinations. The models predicted three levels of severity, and binary performance was assessed to distinguish between depressed or nondepressed states. Sensitivity, specificity, accuracy, and area under the curve (AUC) were measured for binary results. Accuracy was specifically calculated for severity predictions. The five-fold performances underwent the Friedman test, followed by the posthoc Nemenyi test and Cliff δ effect size. A 95% CI was computed using bootstrapping, with the exception of single fold clip prediction AUC, which used normal approximation.

## Results

Demographic characteristics are shown in Table 1 and the findings of clip prediction and the Friedman test are presented in Table 2. The difference among Q&A, MID, and QI (combination of Q&A and MID) is significant in binary sensitivity (*P*=.02), with a large effect (ε^2^=0.47). The differences in binary AUC and extent accuracy are close to significant (*P*=.07 and *P=*.09, respectively), with large effects (ε^2^=0.26 and ε^2^=0.23, respectively). The differences in binary specificity and binary accuracy were not significant and exhibited small effects. Posthoc Nemenyi test results for sensitivity are detailed in Table 3, revealing that Q&A outperformed MID (*P*= .03) with a large effect size (Cliff δ=1.0). The difference between QI and MID is close to significant (*P*=.06) with a large effect (Cliff δ=1.0).

The results of individual prediction and the Friedman test are presented in Table 4. In the RNN voting analysis, the differences among Q&A, MID, and QI were significant in terms of binary sensitivity (*P*<.01) with a large effect (ε^2^=0.61). The differences in binary accuracy and binary AUC were nonsignificant (*P* =.13 and *P=*.09, respectively) but showed large effects (ε^2^=0.18 and ε^2^=0.23, respectively). Posthoc Nemenyi test results on extent accuracy are presented in Table 4.

**Table 1.** Demographic characteristics.

Factors	MDD^a^	nonMDD^b^	*P* value
Sex, n (%)			.13^c^
Male	12 (29.3)	18 (37.5)	
Female	29 (70.3)	30 (62.5)	
Age (years), mean (SD)	38.41 (15.12)	35.98(12.37)	.50^d^
HAM-D^e^, mean (SD)	14.51 (4.66)	–^f^	–
PHQ-9^g^, mean (SD)	13.05 (6.00)	4.17 (2.71)	<.001^d^

a MDD: major depressive disorder.

b nonMDD: non-major depressive disorder.

c Chi-square test was used to derive the *P* value.

d Mann-Whitney U test was used to derive the *P* value.

e HAM-D: Hamilton rating scale for Depression.

f Not applicable.

g PHQ-9: PHQ-9: Patient Health Questionnaire-9.

**Table 2.** Clip prediction results of the MFCC-based^a^ RNN^b^ [29] for paradigm combinations.

Paradigm/statistics/performance	Sensitivity (%), mean (95% CI)	Specificity (%), mean (95% CI)	SA^c^ (%), mean (95% CI)	AUC^d^ (%), mean (95% CI)	EA^e^ (%), mean (95% CI)
Q&A^f^	79.06 (77.06‐83.35)	85.71 (73.30‐90.19)	83.01 (74.43‐86.10)	78.12 (66.11‐82.35)	88.70 (83.15‐91.33)
MID^g^	56.99 (41.78‐63.36)	85.10 (76.36‐89.81)	76.25 (69.90‐80.30)	70.40 (65.09‐73.60)	81.43 (76.42‐85.35)
QI^h^	80.22 (75.88‐84.72)	85.61 (77.06‐89.50)	84.41 (81.98‐86.44)	80.36 (76.45‐82.78)	90.37 (88.81‐91.64)
*P* value^i^	.02	.55	.25	.07	.09
Effect size (ε^2^)	0.47	0.00	0.07	0.27	0.23

a MFCC: mel frequency cepstral coefficients.

b RNN: recurrent neural network.

c SA: screen accuracy.

d AUC: area under the curve.

e EA: extent accuracy.

f Q&A: question-and-answer.

g MID: mental imagery description.

h QI: combination of Q&A and MID.

i Friedman test was used to calculate the *P* value.

**Table 3.** Posthoc test results of the MFCC-based^a^ RNN^b^ clip prediction sensitivity between pairs of Q&A^c^, MID^d^, and QI^e^.

Paradigm statistic item	*P* value^f^	Cliff δ (effect size)
Q&A-MID^g^	.03	1.0 (large)
Q&A-QI^h^	.90	−0.04 (negligible)
QI-MID^i^	.06	1.0 (large)

a MFCC: mel frequency cepstral coefficients.

b RNN: recurrent neural network.

c Q&A: question-and-answer.

d MID: mental imagery description.

e QI: combination of Q&A and MID.

f Nemenyi test.

g Q&A-MID: comparison between Q&A and mental imagery description.

h Q&A-QI: comparison between Q&A and combination of Q&A and mental imagery description.

i QI-MID: combination of Q&A and mental imagery description, and single paradigm mental imagery description.

**Table 4.** Individual prediction results of the MFCC-based^a^ RNN^b^ voting for paradigm combinations.

Paradigm performance	Sensitivity (%), mean (95% CI)	Specificity (%), mean (95% CI)	SA^c^ (%), mean (95% CI)	AUC^d^ (%), mean (95% CI)	EA^e^ (%), mean (95% CI)
Q&A^f^	75.00 (57.50‐87.50)	82.22 (68.89‐86.67)	78.82 (72.94‐83.53)	90.83 (84.17‐95.83)	70.59 (57.65‐77.65)
MID^g^	60.00 (42.50‐67.50)	88.89 (77.78‐95.56)	75.29 (62.35‐80.00)	85.00 (81.94‐90.45)	65.88 (57.65‐71.77)
SQI^h^	75.00 (50.00‐87.50)	88.89 (80.00‐93.33)	82.36 (69.42‐89.42)	92.50 (86.11‐96.11)	80.00 (65.88‐88.24)
*P* value	.29	.26	.13	.09	.009
Effect size	0.04	0.06	0.18	0.23	0.61

a MFCC: mel frequency cepstral coefficients.

b RNN: recurrent neural network.

c SA: screen accuracy.

d AUC: area under the curve.

e EA: extent accuracy.

f Q&A: question-and-answer.

g MID: mental imagery description.

h SQI: combination of conventional questionnaire, Q&A, and mental imagery description.

Table 5. QI outperformed MID (*P*<.05) with a substantial effect (Cliff δ=0.64). The difference between Q&A and QI was nonsignificant (*P*=.14) but indicated a large effect (Cliff δ=−0.48). In the global feature MLP analysis, differences among the paradigms were insignificant and exhibited small effect sizes, with results in Table S3 in Multimedia Appendix 1.

The best fold performance is shown in Table 6. The global feature, SQIV (combination paradigm of CS, Q&A, MID, and VW) MLP achieved a peak individual binary accuracy of 94.12%. Notably, the RNN voting SQI model also achieved a top accuracy of 94.12%, but with a higher extent accuracy of 94.12%, and an AUC of 0.99.

**Table 5.** Post-hoc statistic test results of the RNN^a^ voting individual prediction extent accuracy between pairs of Q&A,^b^ MID^c^, and QI^d^.

Paradigm statistic item	*P* value^e^	Effect size (Cliff δ)
Q&A-MID^f^	.60	0.32 (small)
Q&A-QI^g^	.14	−0.48 (large)
QI-MID^h^	.01	0.64 (large)

a RNN: recurrent neural network.

b Q&A: question-and-answer.

c MID: mental imagery description.

d QI: combination of Q&A and MID.

e Nemenyi test.

f Q&A-MID: comparison between Q&A and mental imagery description.

g Q&A-QI: comparison between Q&A and combination of Q&A and mental imagery description.

h QI-MID: combination of Q&A and mental imagery description, and single paradigm mental imagery description.

**Table 6.** Performance of the best fold of the global feature MLP^a^ SQIV^b^ model, the MFCC-based^c^ RNN^d^ SQI^e^ clip model, and the MFCC-based RNN SQI^e^ voting model.

Method	Paradigm performance	Sensitivity %, (95% CI)	Specificity %, (95% CI)	SA^f^ %, (95% CI)	AUC^g^ (95% CI)	EA^h^ %(95% CI)
MLP^a^	SQIV^b^	100.0 (63.06‐100.0)	88.89 (51.75‐99.72)	94.12 (71.31‐99.85)	0.97 (0.87‐1.0)	76.47 (50.10‐93.19)
RNN^d^	SQI^e^	80.69 (77.74‐83.64)	89.93 (88.60‐91.26)	87.36 (86.09‐88.63)	0.91 (0.90‐0.92)	83.03 (81.60‐84.46)
RNN^d^ voting	SQI^e^	100.0 (63.06‐100.0)	88.89 (51.75‐99.72)	94.12 (71.31‐99.85)	0.99 (0.91‐1.0)	94.12 (71.31‐99.85)

a MLP: multilayer perceptron.

b SQIV: combination of conventional scale, Q&A, mental imagery description, and video watching.

c MFCC: mel frequency cepstral coefficients.

d RNN: recurrent neural network.

e SQI: combination of conventional scale, Q&A, and mental imagery description

f SA: screen accuracy

g AUC: area under the curve

h EA: extent accuracy

The test results of comparison between the RNN voting and the proposed global feature method can be found in Table S4 in Multimedia Appendix 1. The input data are the same in Q&A and MID; for paradigm combinations, we compared RNN-voting QI and the global feature method (ie, combination paradigm of CS, Q&A, and MID; SQI), as they use the most comparable input data. No statistically significant differences were identified between the two methods across all three paradigms. Figures S1-S4 in Multimedia Appendix 1 illustrate the collective and individual learnings of the best MLP SQIV model. The mean attention scores of the features are sequenced by the nonMDD group, the MDD-sub1 group, and the MDD-sub2 group. The complete attention scores with Spearman correlation scores are mentioned in Table S5 in Multimedia Appendix 1, sorted in descending order for the MDD-sub2 group. Previously analyzed nonverbal markers in studies by Fiquer et al [20] and Girard et al [45] can be found in Table S6 in Multimedia Appendix 1. As shown in Figure S1 in Multimedia Appendix 1, the different groups exhibit varying levels of attention to specific features. The Spearman and Kendall correlation coefficients for each feature relative to the target extent of depression are available in Table S5 in Multimedia Appendix 1, where 18 features demonstrated a *P* value<.05.

## Discussion

### Principal Findings

We aimed to evaluate the efficacy of different paradigms via AI on audiovisual signals. We aggregated the four paradigms within the ETD and held 5-fold cross-validation on the two AI models among the paradigm combinations. Our findings show that there are differences in paradigm efficacies, and the AI model learns knowledge consistent with prior human experience.

For the single paradigm with the MFCC-based RNN, Q&A outperformed MID in identifying patients but performed equally in distinguishing extent levels. The difference between Q&A and MID in clip sensitivity was significant, but nonsignificant in individual extent accuracy. This makes Q&A more precise in identifying MDD patients.

For paradigm combinations with the MFCC-based RNN, integrating MID with Q&A slightly decreased clip sensitivity significance but significantly improved individual extent accuracy significance compared with MID. Considering that the difference between QI and Q&A was nonsignificant in either clip sensitivity and extent accuracy, and the differences among Q&A, MID, and QI were nonsignificant in the other performance indexes, we conclude that Q&A demonstrated higher efficacy than MID and suggest that paradigm combinations perform better than a single paradigm. As known, Q&A is a simplified version of a clinical interview, and the questions are all symptom-related, which makes it the most relevant paradigm for MDD and the most important one.

In the individual prediction of the global feature MLP, no significant differences were observed across the paradigm combinations. When fixing the paradigms, no notable differences were found between the MFCC-based RNN voting and the global feature MLP, which validated the global features’ effectiveness. Some large effect sizes were noted, particularly in binary AUC and extent accuracy for Q&A and QI, which may be attributable to feature granularity. The RNN feature integrates both local and global information, with local features benefiting from transfer learning, which enhances performance—achieving a top binary accuracy of 94.12%, a top mean binary accuracy of 82.36%, and a mean extent accuracy of 80.00%. In contrast, the global features may be coarse at the granular level. Even so, the MLP still achieved a mean binary accuracy ranging from 74.12% to 85.88%, with a 95% CI spanning 69.41% to 91.77%, and a top binary accuracy of 94.12%. Overfitting might exist and could result in wide CIs.

Compared with support vector machine models [7], which showed a mean binary accuracy of 78.95% on event-related fMRI [46] and 85.00% on block-related fMRI [47] and CNN [8], which achieved a mean binary accuracy of 85.62% on EEG data, the ETD demonstrated equivalent performance while relying on much more readily accessible daily data. Compared to SRSIs, the audiovisual data are more objective and easier to use. Compared to interview-based assessments such as the HAMD [2], the ETD required approximately 5 minutes, saving about 83% of the time. The ETD’s performance and efficiency support its potential to objectively, accurately, and efficiently screen for MDD.

In the visualization, it is noteworthy that the high- and low-attention features did not intersect, particularly between the MDD group and the nonMDD groups, indicating that participants in different groups exhibited diverse behavioral patterns. Almost all 16 features mentioned by Fiquer et al [20] and Girard et al [21] exhibited high attention scores, with the lowest score being 0.68. Among these, 15 features ranked in the top 20%, except for “head down” which ranked 31st in MDD-sub1, demonstrating consistency with prior studies. Additionally, “head motion velocity” was not included in this study. When comparing with correlation results, 18 features emerged as having significant positive or negative relationships with MDD extent, both in feature items and in correlation trends observed via Spearman and Kendall methods—differing only in specific weights. Of these, 11 aligned with the correlation trends; 5 showed patterns in which the attention scores for the MDD group were either higher or lower than those of the nonMDD group, and only 2 showed no clear trends. For instance, the mean attention score of AU4, interpreted as “frown” by Fiquer et al [20], increased with the extent of MDD, and the Spearman correlation for AU4 was positive (*P*=.005). These high-attention score and correlation-consistent features may serve as urgently needed objective markers and should be further investigated.

The MLP leverages global features representing statistical values throughout the process, sacrificing some detail at the granular level while maintaining low model and computational complexity. Despite potential overfitting, the alignment of the visualization results with correlation findings indicates that the MLP has acquired knowledge consistent with medical prior knowledge, supporting its performance and underscores its potential as a valuable tool. For the inconsistent elements, the neural network introduces significant nonlinearity and captures relationships in high-dimensional spaces. In contrast, Spearman and Kendall correlations are limited to assessing relationships between single inputs and targets. We propose that a trained model can reveal complex multi-input–target relationships that are difficult to define manually. Furthermore, results may vary with the accumulation of additional data.

The ETD’s efficiency—requiring less time and energy— and its objectivity and accuracy make it a flexible and practical tool to be applied across diverse medical scenes that prioritize lightweight and quietness, particularly in screening and health monitoring scenes. Multimodal analysis may produce better results; for instance, AUC of binary depression status increased from 0.72 to 0.76 with networked smartphone sensors combining to text messages [12], and the binary accuracy increased from 76.27% to 95.60% when AU features were added to acoustic features in the baseline MFCC-based RNN [29]. As wearable devices continue to gain popularity, easily obtainable physical signals such as ECG and photoplethysmography can be integrated as additional modalities to enhance clinical outcomes. In our work, we currently use visual, acoustic, and text information jointly, which we believe may be a key point in the high performance observed and should receive more attention in future studies. As audiovisual features are also related to other conditions such as anxiety disorder and schizophrenia [48], or to distinguish between MDD or bipolar depression [49], aggregating multiple paradigms may further improve efficacy.

### Conclusions

The Q&A method showed greater efficacy compared to MID, and combining paradigms may yield better results than using individual paradigms alone. Visualization interpretation showed that the AI method acquired knowledge that aligns with medical expertise and identified several potentially significant markers. By applying AI to multimodal audiovisual signals, these findings position the ETD as a valuable, objective tool for screening MDD and show potential for applications across a broader spectrum of psychiatric disorders with various data modalities.

### Limitations

The efficacy of the modalities remains inadequately explored. Automatically detected AUs may not achieve the reliability of human-labeled results. Additionally, conclusions drawn from current analyses may require revision as sample sizes increase, particularly in deep learning frameworks.

## Supplementary material

10.2196/56057 Multimedia Appendix 1 Definitions, individual prediction results, and attention scores.

## Abbreviations

AI: artificial intelligence
AU: action unit
AUC: area under the curve
CNN: conventional neural network
CS: conventional scale
DL: deep learning
EEG: electroencephalogram
ETD: electronic tool for depression
fMRI: functional magnetic resonance imaging
HAMD: Hamilton rating scale for depression
MDD: major depressive disorder
MFCC: mel frequency cepstral coefficients
MID: mental imagery description
PHQ-8: Patient Health Questionnaire-8
PHQ-9: Patient Health Questionnaire-9
Q&A: question and answering
QI: combination of Q&A and MID
RNN: recurrent neural network
SOTA: state-of-the-art
SQ: combination paradigm of CS and Q&A
SQIV: combination paradigm of CS, Q&A, MID, and VW
SRSI: Self-Report Scales and Inventories
VW: video-watching paradigm
