# Supplementary material for: Comparative Efficacy of MultiModal AI Methods in Screening for Major Depressive Disorder: Machine Learning Model Development Predictive Pilot Study
Source: JMIR Form Res. 2025 May 30;9:e56057. doi: 10.2196/56057 (PMC12143584; doi:10.2196/56057)
Supplement: Multimedia Appendix 1 [file formative-v9-e56057-s001.docx]

**Appendix**

Table S1 Typical AU definition.

| AU | Meaning |
| --- | --- |
| AU4 | Brow Lower, frown in [23] |
| AU6 | Cheek Raiser |
| AU7 | Lid Tightener |
| AU12 | Lip Corner Puller, lips up in [23] |
| AU14 | Dimplier |
| AU15 | Lip Corner Depressor, lips down in [23] |
| AU17 | Chin Raiser |
| AU24 | Lip Pressor, tight lips in [23] |

Table S2 Feature of each modality, “_” means feature name connection.

| Feature | Physical meaning |
| --- | --- |
| AU | same as Tabel 1 |
| au_valid_ratio | frame percentage within the face orientation threshold |
| _read | features during listening/watching process |
| _speak | features during speaking process |
| roll | head rotating angle, head rotating, head to side in [23] |
| pitch | head vertically angel, head up or down in [23], head vertical in [47] |
| yaw | head horizontally angle, head more left or right, head horizontal in [47] |
| gaze_x | distance along x axis, more near or far |
| gaze_y | distance along y axis, more left or right, eye contact in [23] |
| gaze_z | distance along z axis, more up or down, eye contact in [23] |
| mean | mean of value, big or small |
| std | standard variance, dispersion of data distribution, dispersive or converge |
| mad | Median Absolute Deviation, more robust dispersion of data distribution, dispersive or converge |
| kurt | kurtosis, flatness of data distribution, dispersive or converge |
| MFCC | frequency coefficients, physical meaning not clear |
| d_delta | double delta |
| audio_duration | pure time length when speaking, willingness to express, silence in [23] |
| text_sentimental_score | emotion more happy or sad |

Table S3 Individual prediction results of global feature MLP for paradigm combinations.

| Paradigm\Performance | SENS (%), mean(95%CI) | SPEC (%), mean(95%CI) | SA (%), mean(95%CI) | AUC (%), mean(95%CI) | EA (%), mean(95%CI) |
| --- | --- | --- | --- | --- | --- |
|  |  |  |  |  |  |
| CS | 67.50 (62.50-72.50) | 80.00 (71.11-84.45) | 74.12 (69.41-84.71) | 80.28 (65.83-85.00) | 62.35 (55.29-65.89) |
| Q&A | 90.00 (80.00-95.00) | 80.00 (53.33-91.11) | 84.71 (70.59-88.24) | 76.11 (60.84-83.05) | 68.23 (57.64-76.47) |
| MID | 85.00 (67.50-95.00) | 82.22 (71.11-91.11) | 83.53 (78.82-85.89) | 77.22 (63.06-86.39) | 62.35 (55.29-68.24) |
| VW | 62.50 (52.50-67.50) | 95.56 (88.89-97.78) | 80.00 (76.47-81.17) | 73.33 (61.94-81.66) | 64.71 (61.17-67.06) |
| SQ | 82.50 (57.50-95.00) | 86.67 (77.78-93.33) | 84.71 (75.29-89.42) | 82.50 (69.44-92.22) | 63.53 (56.47-68.24) |
| SQI | 85.00 (75.00-92.50) | 84.45 (73.34-91.11) | 84.71 (78.82-89.42) | 81.94 (76.39-89.44) | 64.71 (64.71-64.71) |
| SQIV | 82.50 (57.50-92.50) | 88.89 (71.11-95.56) | 85.88 (78.82-91.77) | 82.22 (72.22-91.11) | 67.06 (62.35-71.77) |
| ^a^*P* value | .18 | .23 | .31 | .83 | .97 |
| ^b^Effect size | 0.10 | 0.08 | 0.04 | 0.00 | 0.00 |
| *P* value^a^ | Friedman test | Effect size^b^ | Epsilon square |  |  |

Table S4 Statist test results between the RNN voting and the proposed global feature method on individual prediction.

| Paradigm | statistics\  Performance | SEN (%), mean(95%CI) | SPE (%), mean(95%CI) | SA (%), mean(95%CI) | AUC (%), mean(95%CI) | EA (%), mean(95%CI) |
| --- | --- | --- | --- | --- | --- | --- |
|  |  |  |  |  |  |  |
| Q&A | ^a^*P* value | .46 | .79 | .47 | .13 | .19 |
|  | ^b^Effect size | 0.24 | 0.16 | 0.40 | 0.68 | 0.48 |
| MID | ^a^*P* value | .06 | .44 | .13 | .06 | 1.00 |
|  | ^b^Effect size | -0.96 | 0.2 | -0.68 | 0.52 | -0.16 |
| QI/SQI | ^a^*P* value | .63 | .63 | 1.00 | .13 | .13 |
|  | ^b^Effect size | -0.28 | 0.36 | 0.08 | 0.76 | 0.76 |
| *P* value^a^ | Wilcoxon Signed-Rank test | Effect size^b^ | Cliffs delta |  |  |  |


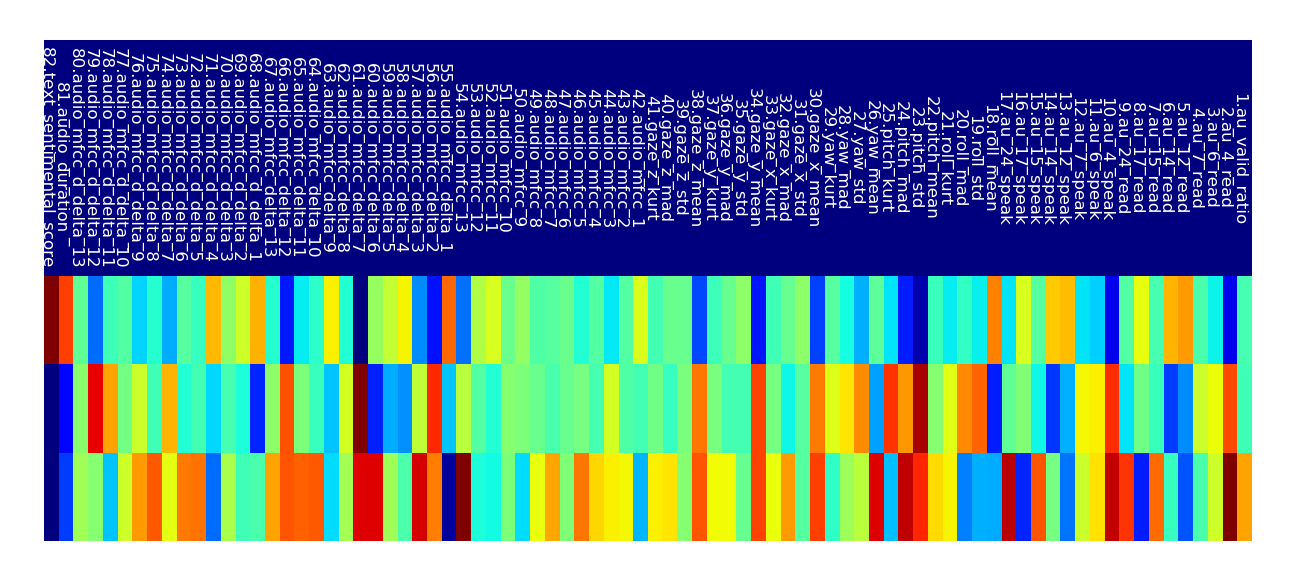


Figure S1 Average attention scores of the best global feature SQIV MLP model on the non-MDD group, MDD-sub1 group, and MDD-sub2 group.
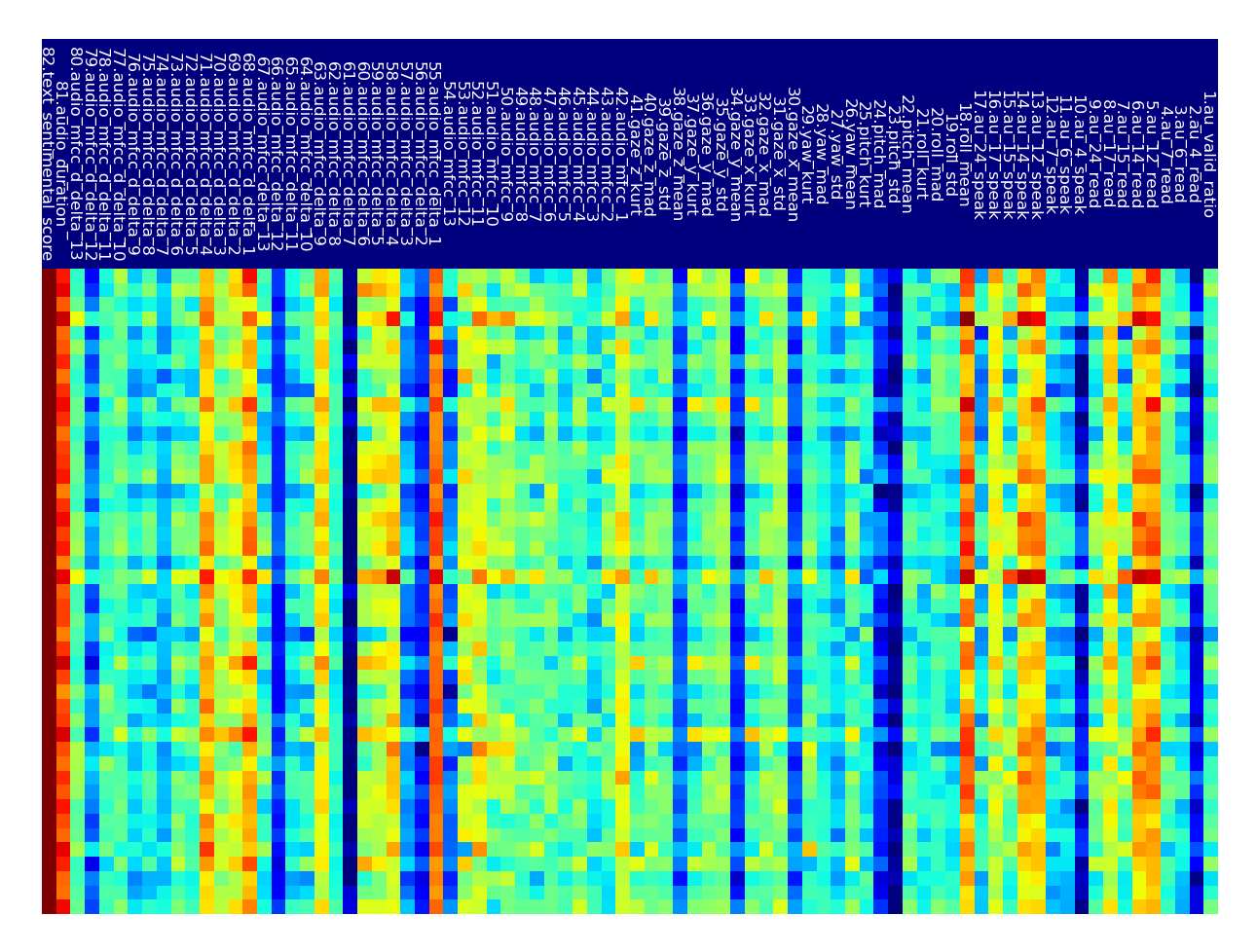


Figure S2 Attention score of the best global feature SQIV MLP model on each participant of the non-MDD group.


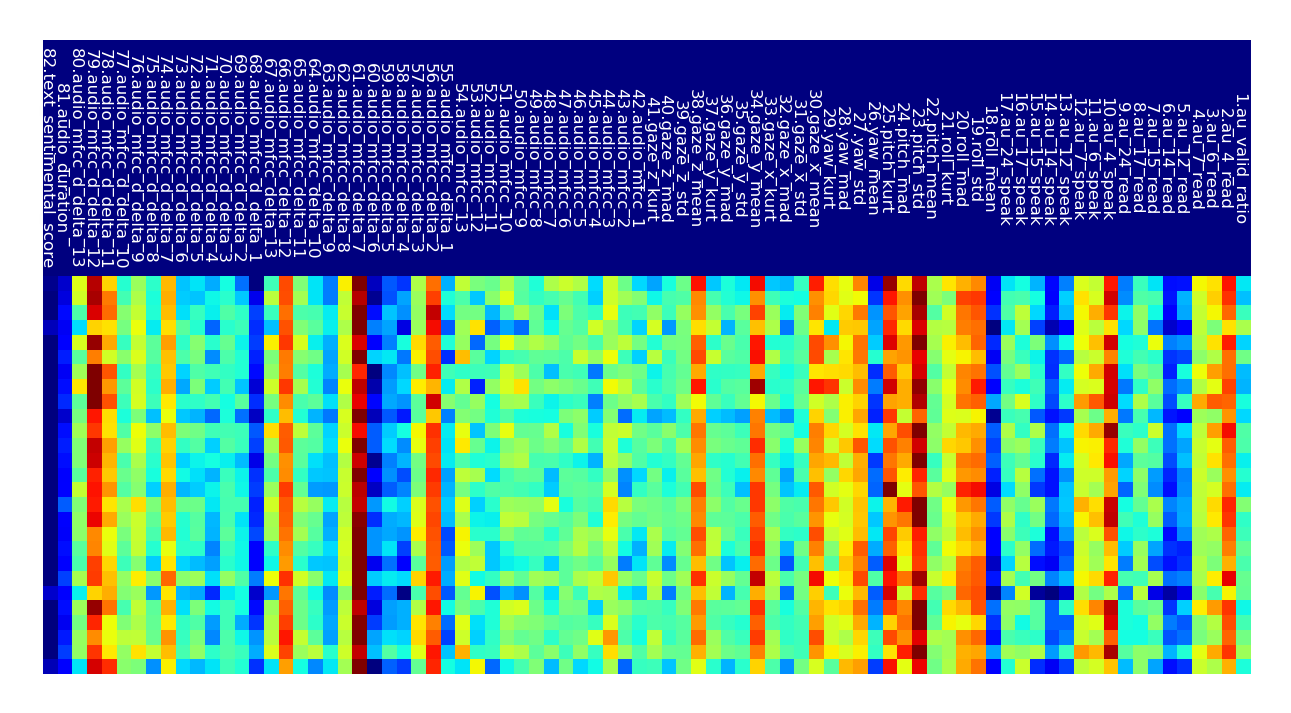


Figure S3 Attention score of the best global feature SQIV MLP model on each participant of the MDD-sub1 group.


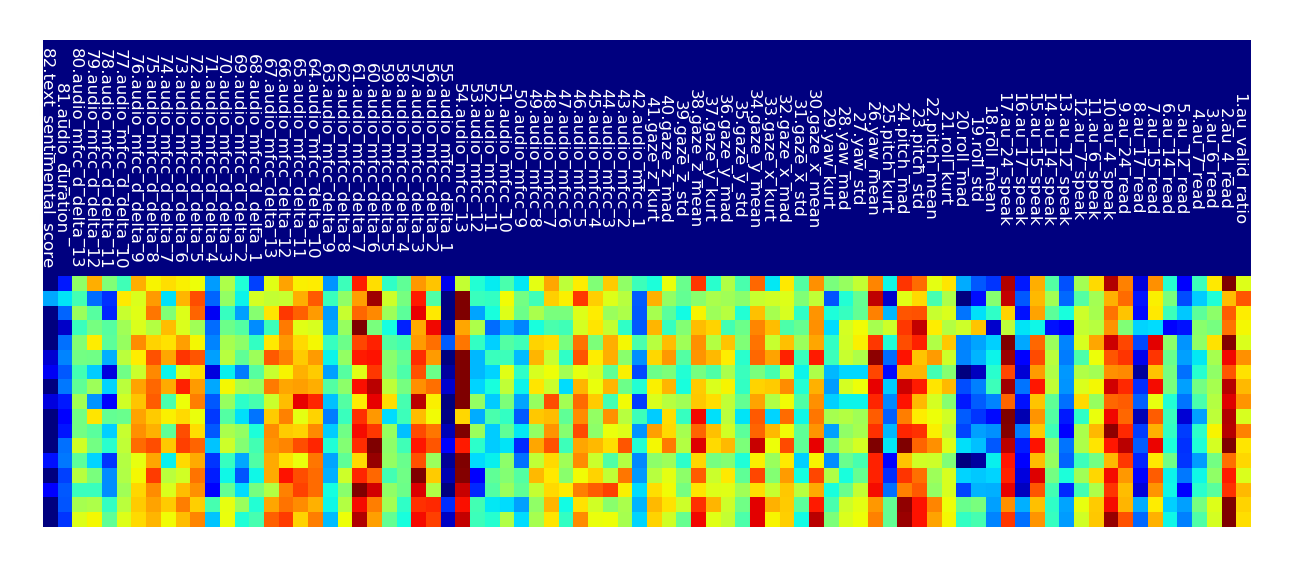


Figure S4 Attention score of the best global feature SQIV MLP model on each participant of the MDD-sub2 group.

Table S5 Whole attention scores with Spearman correlation and *P* value and sorted by MDD-sub2 group’s attention score in descending order.

| Index | Healthy | Mild | Moderate or severe | Spearman  correlation score | *P* value |
| --- | --- | --- | --- | --- | --- |
| 2 | 0.0942 | 0.8297 | 1.0000 | 0.2351 | .005 |
| 54 | 0.2348 | 0.5716 | 0.9968 | 0.1557 | .07 |
| 17 | 0.3486 | 0.4682 | 0.9470 | -0.1142 | .17 |
| 24 | 0.1549 | 0.7465 | 0.9417 | 0.1019 | .23 |
| 10 | 0.0954 | 0.8614 | 0.9388 | 0.2380 | .004 |
| 57 | 0.2635 | 0.5825 | 0.9257 | 0.0475 | .58 |
| 61 | 0.0000 | 1.0000 | 0.9165 | 0.2760 | <.001 |
| 26 | 0.4619 | 0.2839 | 0.9158 | 0.0826 | .33 |
| 60 | 0.5339 | 0.1567 | 0.9148 | 0.1147 | .18 |
| 23 | 0.0409 | 0.9628 | 0.8703 | 0.1224 | .15 |
| 9 | 0.4508 | 0.3485 | 0.8550 | -0.1486 | .08 |
| 30 | 0.1882 | 0.7777 | 0.8433 | 0.1487 | .08 |
| 34 | 0.1483 | 0.8382 | 0.8389 | 0.1551 | .07 |
| 66 | 0.1499 | 0.8231 | 0.8197 | 0.1274 | .13 |
| 15 | 0.4533 | 0.3672 | 0.8174 | -0.0360 | .67 |
| 38 | 0.1961 | 0.7833 | 0.8174 | 0.1510 | .07 |
| 64 | 0.4032 | 0.4183 | 0.8157 | 0.0566 | .51 |
| 75 | 0.3946 | 0.4286 | 0.8135 | 0.1335 | .11 |
| 65 | 0.3574 | 0.5031 | 0.8082 | 0.0463 | .59 |
| 7 | 0.4346 | 0.4184 | 0.7939 | -0.0686 | .42 |
| 72 | 0.4179 | 0.4315 | 0.7867 | -0.0411 | .63 |
| 46 | 0.3922 | 0.4942 | 0.7822 | 0.2020 | .02 |
| 73 | 0.4536 | 0.3928 | 0.7776 | 0.0660 | .44 |
| 56 | 0.1423 | 0.8653 | 0.7751 | -0.0006 | .99 |
| 76 | 0.3333 | 0.5971 | 0.7441 | 0.1264 | .14 |
| 32 | 0.4757 | 0.3700 | 0.7430 | 0.1295 | .13 |
| 1 | 0.4348 | 0.4258 | 0.7379 | -0.0429 | .61 |
| 67 | 0.3982 | 0.5222 | 0.7347 | 0.0318 | .71 |
| 48 | 0.4562 | 0.4397 | 0.7306 | 0.1072 | .21 |
| 45 | 0.4509 | 0.4348 | 0.6804 | -0.1926 | .02 |
| 22 | 0.4200 | 0.5134 | 0.6792 | -0.0754 | .37 |
| 11 | 0.3301 | 0.6556 | 0.6705 | 0.1339 | .11 |
| 40 | 0.4804 | 0.4157 | 0.6675 | 0.1107 | .19 |
| 44 | 0.3526 | 0.6093 | 0.6558 | 0.1687 | .05 |
| 41 | 0.4323 | 0.4870 | 0.6531 | 0.2010 | .02 |
| 21 | 0.3584 | 0.6211 | 0.6493 | 0.1249 | .14 |
| 43 | 0.4516 | 0.4465 | 0.6447 | -0.0981 | .25 |
| 36 | 0.4760 | 0.4303 | 0.6433 | 0.0688 | .42 |
| 37 | 0.4242 | 0.4991 | 0.6407 | 0.2062 | .01 |
| 49 | 0.4482 | 0.4793 | 0.6360 | 0.0463 | .59 |
| 33 | 0.4294 | 0.4960 | 0.6341 | 0.1929 | .02 |
| 74 | 0.2961 | 0.7187 | 0.6288 | 0.0635 | .46 |
| 3 | 0.3647 | 0.6398 | 0.6008 | 0.1332 | .12 |
| 77 | 0.4510 | 0.4919 | 0.5996 | -0.0906 | .29 |
| 12 | 0.3480 | 0.6484 | 0.5963 | -0.1712 | .04 |
| 27 | 0.3006 | 0.7591 | 0.5775 | -0.0053 | .95 |
| 70 | 0.5269 | 0.4375 | 0.5581 | 0.0560 | .51 |
| 62 | 0.3921 | 0.6073 | 0.5471 | 0.0389 | .65 |
| 80 | 0.4654 | 0.5177 | 0.5436 | -0.0368 | .66 |
| 59 | 0.5862 | 0.2964 | 0.5431 | -0.0567 | .50 |
| 28 | 0.3767 | 0.6655 | 0.5416 | -0.0795 | .35 |
| 39 | 0.4748 | 0.4979 | 0.5148 | -0.0801 | .35 |
| 79 | 0.2319 | 0.9090 | 0.5127 | 0.0791 | .35 |
| 47 | 0.4791 | 0.4727 | 0.5076 | -0.0701 | .41 |
| 51 | 0.4788 | 0.5149 | 0.5036 | 0.0415 | .63 |
| 14 | 0.6946 | 0.1790 | 0.4929 | -0.1927 | .02 |
| 35 | 0.5210 | 0.4334 | 0.4763 | -0.0742 | .38 |
| 31 | 0.5233 | 0.4563 | 0.4569 | -0.0885 | .30 |
| 58 | 0.6505 | 0.2668 | 0.4444 | 0.0114 | .89 |
| 68 | 0.7211 | 0.1618 | 0.4395 | -0.1383 | .10 |
| 4 | 0.4388 | 0.5970 | 0.4385 | -0.1436 | .09 |
| 69 | 0.5977 | 0.3832 | 0.4280 | -0.0346 | .68 |
| 6 | 0.7169 | 0.1873 | 0.4179 | -0.1621 | .05 |
| 29 | 0.4543 | 0.6207 | 0.4085 | 0.1393 | .10 |
| 53 | 0.5653 | 0.4494 | 0.3840 | -0.0318 | .71 |
| 52 | 0.6101 | 0.3917 | 0.3730 | -0.3378 | <.001 |
| 63 | 0.6546 | 0.3188 | 0.3423 | -0.0793 | .35 |
| 50 | 0.5297 | 0.5010 | 0.3418 | -0.1052 | .22 |
| 78 | 0.4231 | 0.7351 | 0.3188 | 0.2368 | .005 |
| 25 | 0.3488 | 0.8528 | 0.3135 | -0.1471 | .08 |
| 42 | 0.6145 | 0.4304 | 0.3028 | -0.2635 | .002 |
| 19 | 0.3632 | 0.8043 | 0.2997 | 0.1510 | .07 |
| 18 | 0.7713 | 0.1538 | 0.2945 | -0.2515 | .003 |
| 20 | 0.4116 | 0.7624 | 0.2536 | 0.0769 | .37 |
| 13 | 0.7104 | 0.2993 | 0.2385 | -0.0921 | .28 |
| 71 | 0.7135 | 0.3371 | 0.2311 | 0.0691 | .42 |
| 5 | 0.7437 | 0.2626 | 0.2077 | -0.0880 | .30 |
| 81 | 0.8414 | 0.1325 | 0.1853 | -0.1714 | .04 |
| 16 | 0.6096 | 0.5049 | 0.1623 | -0.0396 | .64 |
| 8 | 0.6290 | 0.4853 | 0.1529 | -0.0169 | .84 |
| 55 | 0.7978 | 0.3174 | 0.0261 | -0.2750 | <.001 |
| 82 | 1.0000 | 0.0000 | 0.0000 | -0.5065 | <.001 |

Table 6 Nonverbal features listed in prior work [46][47] and the corresponding attention scores in each group.

| Nonverbal feature | Corresponding feature | Attention score in the non-MDD group | Attention score in the MDD-sub1 group | Attention score in the MDD-sub2 group |
| --- | --- | --- | --- | --- |
|  |  |  |  |  |
| frown | au4 read | 0.0942 | 0.8297 | 1.0000 |
| frown | au4 speak | 0.0954 | 0.8614 | 0.9388 |
| lips down | au15 read | 0.4346 | 0.4184 | 0.7939 |
| lips down | au15 speak | 0.4533 | 0.3672 | 0.8174 |
| head down | pitch mean | 0.4200 | 0.5134 | 0.6792 |
| head to side | roll mean | 0.7713 | 0.1538 | 0.2945 |
| eye contact | gaze y mean | 0.1483 | 0.8382 | 0.8389 |
| eye contact | gaze z mean | 0.1961 | 0.7833 | 0.8174 |
| au 12 | au12 read | 0.7437 | 0.2626 | 0.2077 |
| au 12 | au12 speak | 0.7104 | 0.2993 | 0.2385 |
| au 14 | au14 read | 0.7169 | 0.1873 | 0.4179 |
| au 14 | au14 speak | 0.6946 | 0.1790 | 0.4929 |
| au 24 | au24 read | 0.4508 | 0.3485 | 0.8550 |
| au 24 | au24 speak | 0.3486 | 0.4682 | 0.9470 |
| vertical amplitude | pitch std | 0.0409 | 0.9628 | 0.8703 |
| horizontal amplitude | yaw std | 0.3006 | 0.7591 | 0.5775 |
